# The Sociogenomics of Social Stratification and General Theories of Inequality

**DOI:** 10.1111/1468-4446.70138

**Published:** 2026-06-10

**Authors:** Herman G. van de Werfhorst

**Affiliations:** ^1^ European University Institute Fiesole Italy

**Keywords:** genes, inequality, social stratification, social theory

## Abstract

The field of the sociogenomics of inequalities would benefit from discussing what its findings and ambitions mean for core theories of social stratification and mobility. Do sociologists need to reconsider their critique to structural‐functionalist theory of stratification, and its neo‐classical economic allies, that emphasise efficient sorting of individuals to social positions? Or do their interpretations of genetic correlates of inequality need to include reference to constructivist processes that have defined the genetic mix that has come to be rewarded in a society? I argue that, with the current sociogenomics methods, it is hard to disentangle these theories, which are however fundamentally different regarding the nature and structure of social stratification. Using simulations that distinguish context‐dependent from context‐independent genetic variability, it is shown that the field always needs to make untestable assumptions about the nature of genetic differences.

## 1 Introduction

The sociogenomics of social stratification is a rapidly developing field that generates knowledge about the relations between genes, environments and social stratification outcomes like educational attainment or earnings. Two important hypotheses in the field concern gene‐environment interactions (GxE), and genetic nurturing (Isungset et al. 2022). The GxE hypothesis holds that the genetic component of stratification outcomes is larger if social barriers to educational and economic performance are removed. Studies have looked at, for example, family‐level resources and state‐level public policies as barrier‐removing, and tested whether these reduce the relevance of the environment, and increase the relevance of genes for the determination of educational attainment, earnings, and social mobility (Engzell and Tropf 2019; Rimfeld et al. 2018).^1^ The genetic nurturing hypothesis states that parental genes have a direct association with children's stratification outcomes independent of children's genes (Kong et al. 2018). Parental genes can, for instance, translate into an environment that is conducive to children's educational attainment.

Both hypotheses emphasise the relevance of social context. It is not surprising that social scientists are increasingly embarking upon this field, as their theories may be helpful for geneticists to understand why context matters, and which additional specific hypotheses can be derived (Ghirardi and Bernardi 2025). But the implications of findings from sociogenomics for sociological theories need more thought.

There is a lot of awareness among geneticists and social scientists about the possible limitations and pitfalls of the proposed designs. Most importantly, genetic correlates do not show causal effects, so we should be careful with an essentialist understanding of the genetic mix that is apparently correlated with social stratification (Turkheimer 2019; Abdellaoui et al. 2025). Nevertheless, the field now increasingly uses within‐family designs exploiting genetic data of siblings, reducing the gene‐trait correlations substantially on social stratification outcomes like cognitive ability and educational attainment (Morris et al. 2020). Statistical models are developed to isolate ‘causal effects’ of genes, neutralizing population stratification (Bulik‐Sullivan et al. 2015). Remaining correlations between genes and stratification outcomes may suggest that the within‐family effects reflect ‘something real’ in the individual genetic variation concerning the placement in the social structure. Thus, the better our understanding of the context‐dependency of the working of genes, the more we may think that, under certain conditions, the remaining genetic variation reflects something essential in humans that predicts their likelihood to do well in school or the labour market.

While the field is rightfully cautious about essentialist interpretations of genes in relation to stratification, it is important to discuss the ramifications of the findings of sociogenomics for broader sociological theories of inequality. If research were able to eliminate confounding factors like assortative mating or population stratification, or define environmental factors that increase the genetic contribution to stratification, there may indeed be ‘real’ individual variation on which basis the social order is (partially) determined. Such an interpretation connects strongly to rational‐functional allocation models that state that modern society sorts individuals to positions based on efficiency and scarcity. This model finds its social scientific origin in a structural‐functionalist explanation of social stratification (Davis and Moore 1945), that sees meritocratisation (i.e., the tendency to allocate people to positions increasingly based on their merits rather than their environmental factors) as part of a broader modernisation process towards rationalisation and efficiency. Similarly, geneticists have argued that ‘the genetic influence on these social outcomes [educational attainment and occupational status] can be viewed as an index of success in achieving meritocratic values of equality of opportunity’ (Rimfeld et al. 2018). Formulated this way, structural‐functionalism has clear connections to neo‐classical economics, a field that similarly assumes that earnings inequalities are a product of supply of and demand for skills and cognitive ability.

However, this interpretation of the world stands in stark contrast to sociological theories of social closure, that deny that the allocation of individuals to structural positions follows a model of efficiency (Van de Werfhorst and Solga 2026). Instead, closure theories argue, social stratification results from informal and formal criteria for selection into prestigious positions that are not employed to enhance efficient sorting, but are meant to keep social circles closed to insiders (read: advantaged groups) and opportunities limited to outsiders (disadvantaged groups). Theories of social closure, mostly inspired by Max Weber (Weber 1922), come in different kinds, including institutional perspectives on the regulated access to occupations through certification and licencing (Weeden 2002), theories defining informal preferences for certain cultural status backgrounds (Rivera 2012), neo‐institutionalist accounts stipulating mythical beliefs in education as a governing principle in the world society (Baker 2014), and cultural perspectives of the valuation of performance (Lamont 2012). A common thread in all these arguments is that the appreciation of individuals in schools and work places is not (only) following a rational‐functional logic of efficiency and rationality, but must (also) be seen as resulting from a social construction of the world we live in, in which powerful groups are able to define the rules of the game. Neo‐classical and closure perspectives are complementary theories of stratification—they can both be true to a certain extent, with regard to some processes rather than others, and/or more applicable in one context rather than another (Van de Werfhorst 2011).

Importantly, closure perspectives may not only explain the role of context, either as a confounder or moderator, but may also be underlying the roles of genes themselves. A social closure perspective on genetic correlates to social stratification would hold that the appreciation of a specific genetic mix of individuals (such as scoring high on the polygenic score for educational attainment), should be seen as a product of societal construction, with the explicit aim to restrict access to prestigious positions to outsiders. As an illustration, early sociological scholarship has mentioned the red hair example, a thought experiment that if society had persistently denied people with red hair to obtain education, the genes predictive of red hair would have ended up in the genetic mix that predicts educational attainment (Jencks et al. 1972). It is a societal choice to give preference to certain populations (i.e., there is population stratification), which could lead to misleading conclusions about group differences in genetic predispositions. A constructivist account of the reward of a particular genetic markup, exemplified in stratification‐relevant traits like intelligence, can be seen in many different corners of the social sciences. In discussing the role of ability for economic inequalities, famous economist of economic inequality the late Anthony Atkinson wrote that ‘the distribution [of ability] depends on the measurement rod used and cannot be defined independently of it’. (Atkinson 1975). From a more radical perspective, the concept of intelligence has been criticised as a tool to justify the intergenerational reproduction of advantage (Bourdieu 1993).

With these two discourses at hand—an essentialist versus a constructivist interpretation of the role of genes—what can we learn about sociological theories of stratification? It is possible that scholarship finds real relevant differences among individuals in the ability to deal with complex information, and liberal‐democratic societies tend to efficiently select and sort on this basis. But that would imply that rational‐functional models of stratification are more credible than sociological scholarship has believed in the past half century. The rational‐functional model has been refuted, because it is teleological (the rewarded traits must naturally be the ones that have become more relevant), tautological (it defines what is rewarded in society by looking at what is rewarded in society), and empirically incorrect (socioeconomic background maintains to have a major impact on children's attainments). Are these critiques still valid if there is a ‘real’ difference between individuals concerning their ability to deal with complex information?

However, if one adheres to a social‐constructivist explanation for the workings of genes, the field of sociogenomics needs to be more careful in the interpretation of its findings. The field may want to study how societies have come to adopt a particular social ordering in which apparently certain genetic predispositions matter. Such an endeavour would be part of a broader agenda that questions how merits are actually defined. Does the definition of merit (and the ways to measure it) result from power differences between groups? To develop the debate into a positive, rather than normative one, it would be a useful contribution of the social sciences if they were able to find empirical tests of such claims.

## 2 A Simulation Model

Given the inescapable contextual dependence of the valuation of the genetic makeup, it is useful to do simulations.^2^ Specifically, one can simulate several scenarios with regard to the ‘real’ (or essentialist) versus ‘cultural’ (or constructivist) component of measured genes (such as measured in the workhorse of contemporary scholarship on the genetics of inequality: the Polygenic Index for education or socioeconomic attainment, *PGI*), and how these components shape the social mobility process from socioeconomic origin to socioeconomic destination. The simulation works as follows. Two latent components are generated independently: the real component (*REAL*) refers to the genetic variation that would lead to advantage equally in all human societies (or even on a desolate island, for instance when smart people are better able to use complex information to ensure survival), while the cultural component (*CULT*) refers to a cultural bias in what has ended up in the genetic markup by processes such as population stratification or assortative mating. Both components are continuous standardised variables generated for a sample of *N* = 1000 individuals. In both worlds, social origin is correlated with the cultural component (*r* = 0.40) but not, or only weakly, with the real component (see below). The observable *PGI* is then constructed as a weighted combination of *REAL* and *CULT* plus noise, with the weights varying across scenarios (see below). Crucially, socioeconomic destination is generated as a function of the *PGI* and social origin directly—not of *REAL* and *CULT* separately. This is a key design choice: it means that both the real and the cultural component of the *PGI* drive destination equally in all scenarios, entirely through their combined effect on the *PGI*. What varies across scenarios is only the proportion of the *PGI* that is ‘made up of’ real versus cultural variation.

One extreme position would be that all that is observed in a *PGI* is culturally laden, and there is no component in it that would give real advantage in all human societies or on desolate islands. Or formally, adding a random error component, PGI=0∗REAL+1∗CULT+ε, *scenario 1*. This position is similar to the Bourdieusian critique to the concept of intelligence. In that case, the *PGI* would be fully predicted by the cultural component of it, and not by some real context‐independent component. Another extreme position would have a fully essentialist understanding of *PGI*, which may be the kind of variation that the sociogenomics field is after when they try to isolate genetics effects from cultural biases (although the field is aware that they cannot isolate this real variation). In this extreme position, the cultural component of the *PGI* is equal to zero. Or, PGI=1∗REAL+0∗CULT+ε, *scenario 4*. Two mixed positions would be that *PGI* includes both a real and a cultural component, with either a larger fraction of the cultural (PGI=0.3∗REAL+0.7∗CULT+ε, *scenario 2*), or of the real component, (PGI=0.7∗REAL+0.3∗CULT+ε, *scenario 3*). Altogether I thus create four scenarios that vary in the existence and balance of real and cultural components of the polygenic index score.

**FIGURE A1:**
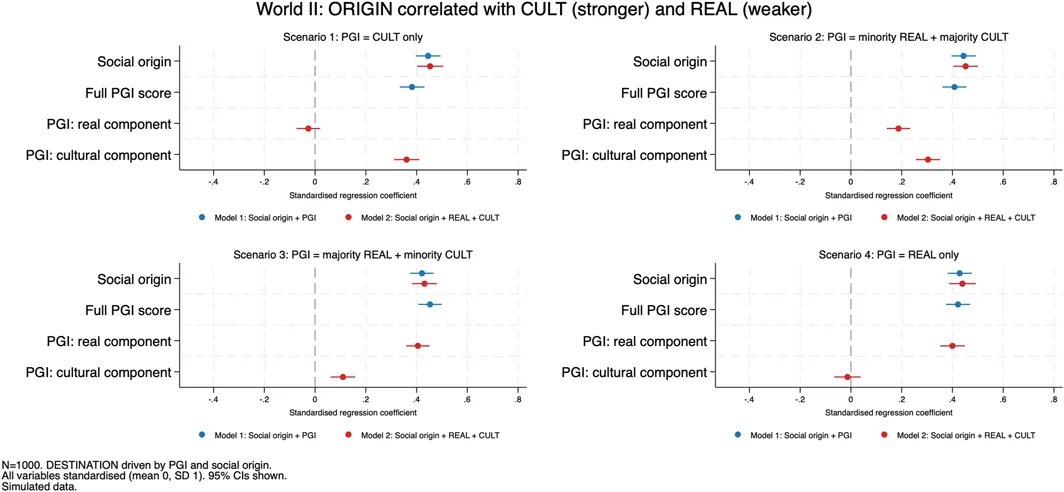
OLS regression coefficients predicting socioeconomic destination (assuming a correlation between social origin and the ‘real’ component of PGI).

Then, there are two more variations, depending on whether social origin is correlated to the real component of the genetic markup or not. In *World I*, social origin is predictive of the cultural component of the *PGI* (*r* = 0.40), but not of the real component (*r* = 0). This represents a world in which social origin shapes the cultural traits that happen to be rewarded, but in which the context‐independent genetic propensity to perform well is randomly distributed across social classes. In *World II*, social origin predicts both the cultural and real components (*r* = 0.40 and *r* = 0.20 respectively), reflecting a world in which even the context‐independent genetic capacity is unevenly distributed across social classes, for instance due to long‐run assortative mating within socioeconomic strata. The fact that results are very similar across these two worlds is itself informative: it shows that the key question is not whether the real genetic component is itself socially stratified, but whether the measured *PGI* can be interpreted as representing ‘something real’. Because the results are very similar across the two worlds, the results for World II are provided in the Appendix (Figure A1).

In all contexts, socioeconomic origin has a direct effect on socioeconomic destination, reflecting the well‐known finding that social mobility processes have direct effects of origin controlling for achievements and attainments (Bernardi and Ballarino 2016; Friedman and Laurison 2020). With these definitions, we can then estimate intergenerational mobility models, for each of the four scenarios by the two worlds, yielding eight simulated contexts. See Table 1 for the eight contexts.

**TABLE 1:** Eight simulated contexts of social stratification and mobility.

		Scenario 1	Scenario 2	Scenario 3	
		PGI is only cultural	PGI is a bit more cultural than essentialist	PGI is a bit more essentialist than cultural	Scenario 4 PGI is only essentialist
World 1	Only the cultural component of the PGI is correlated with social origin	1	2	3	4
World 2 (appendix)	Both the cultural and essentialist component of the PGI are correlated with social origin	5	6	7	8

For each simulated context we estimate two regression models. Model 1 mimics the existing literature by estimating an effect of *PGI* and social origin on destination, without separating the cultural and real components of *PGI*. Model 2 instead uses the latent *REAL* and *CULT* components directly as separate predictors alongside social origin—a separation that is not possible with contemporary techniques, since these latent components are never observed in real data. Model 2 thus shows what one would find in an ideal (counterfactual) world in which the two components of genetic variation could be disentangled.

**FIGURE 1:**
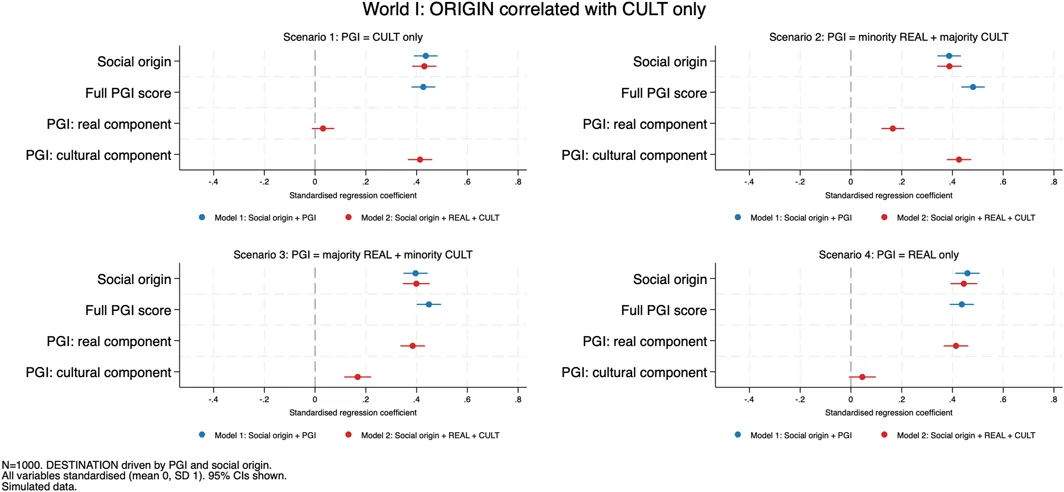
OLS regression coefficients predicting socioeconomic destination.

Figure 1 shows the regression coefficients of two models, for four scenarios in World I. Results for World II, in which a correlation between social origin and the real component of the PGI is assumed, are highly similar and shown in the Appendix.

The effects of social origin and the full PGI score (Model 1) are similar across the four scenarios, as expected: the total predictive power of the PGI for destination is held constant by construction, since destination is always driven by the composite PGI with the same coefficients regardless of scenario. More important is what happens when the two components are separated (Model 2). The more the PGI is composed of the cultural component, the larger is the coefficient on CULT and the smaller on REAL in Model 2; when the PGI is purely cultural (Scenario 1), the real component has no predictive power for destination because it contributes nothing to the PGI that drives destination. The mirror holds: when the PGI is purely real (Scenario 4), the cultural component carries no independent predictive weight. The intermediate scenarios show that the estimated coefficients on the two components reflect their weights in the PGI, not any independently varying relationship with destination. This illustrates the core identification problem: because the two latent components always enter destination only through their combined effect on the measured PGI, no empirical strategy working with the PGI alone can recover their separate contributions.

These results may seem obvious, they clarify that, as long as the sociogenomics field is unable to separate the real from the cultural component of what ended up in the genetic mix that is appreciated in education and labour markets, it remains unknown to what extent the field finds ‘something real’ in the stratification of contemporary societies. Within‐family designs, or statistical models that filter out population stratification, or an expansion of genetics data from other populations than European ancestry, will not solve this problem. Countries across the world have moved towards a rather standardised way of educating and rewarding workers (Schofer and Meyer 2005; Bromley 2010), and similar cultural biases coded in the genetic markup may be persistent everywhere. As a group of behavioural geneticists so clearly summarise, there is always the assumption of a meritocratic society underlying the sociogenomics of socioeconomic status (Abdellaoui et al. 2025).

## 3 Concluding Remarks

As argued above, with the advancement of the field of the sociogenomics of stratification, we need to think of the ramifications of the findings for broad sociological theories of stratification. Genes are associated with outcomes like educational or occupational attainment, and the more precise estimates of genetic correlates to stratification may suggest that there is a context‐independent part of the genetic markup that taps into an essentialist understanding of inequalities. However, as the field realises, it is not easy, if not impossible, to draw conclusions on context independence. In fact, as the field rightfully acknowledges, empirical genetics research must always work with the assumption that a society has come to appreciate the particular genetic mix (Akimova et al. 2025; Abdellaoui et al. 2025), inducing a cultural component into the polygenetic scores. There is evidence that the cultural component is in fact rather large. Heritability estimates are much lower using GWAS compared to twin models (that compare the similarity of identical and fraternal twins) (Tropf et al. 2017). Furthermore, models that estimate heritability to vary across contexts show that a lot of heterogeneity in the heritability of education, assuming that the context‐independent part is small (Tropf et al. 2017). But even the context‐independent part cannot be equated with the ‘real’, essentialist component of genes, as there could be similarity of the cultural appreciation of particular traits.^3^

Therefore, this note discussed the assumption that there is some underlying variability among individuals that would correlate with success in life in all human societies; a measure of context‐independent genetic variation in the propensity to do well in obtaining scarce resources. One could see the improvements in the behavioural genetics field as an attempt to get to context‐independent, random variation in genetic markup.

Whether this assumption is valid is not the point here, but the field is at least suggestive of the existence of ‘something real’ even if contemporary research is not able to measure context‐independent variation. I simulated data to create this context‐independent genetic propensity to do well, and added variation that enables cultural elements to the measured genetic markup.

The results of the simulations are highly dependent on assumptions made. In a scenario in which society has come to appreciate the culturally‐induced component of what we genetically observe, a focus on the correlates of measured genes may be misguided in thinking that ‘something real’ has been found. It may be more preferable to think of measured genes as representing the circumstances with which one is equipped, and as it goes with circumstances, they often have an important cultural component.

Reflecting on the four different scenarios that were simulated, the main axis was not whether the context‐independent part of genes is socially stratified. What was the main axis of difference across these imaginary worlds is the extent to which measured genes represent a constructivist or essentialist component. And this matter comes down to fundamental differences between core sociological theories of efficient sorting or social closure. It would be good if social‐scientific contributions to the field would make such connections to sociological theories explicit, including the difficulty in making these.

## Funding

The authors has nothing to report.

## Conflicts of Interest

The author declares no conflicts of interest.

## Data Availability

This paper only uses simulated data. Replication files for the simulations are available at https://osf.io/efxhd/.

## Endnotes

^1^
With today's dominant technique of Genome‐Wide Association Studies (GWAS), it is, it should be noted, hard to find gene‐environment interactions because the GWAS finds the general genetic propensity for outcomes averaged over a large number of contexts (Conley 2017).

^2^
I used Claude version 1.9659.2 to create AI‐assisted human‐supervised Stata code to produce the simulations. Code is available on https://osf.io/efxhd/.

^3^
On top of that, there is a fundamental uncertainty in human behaviour that makes it impossible to reduce error to zero, even in a world with improved data and methods (Yan and Rahal 2025). This may make it hard to test for essentialist hypothesis anyway.
